# Hypoxia‐responsive miR‐124 and miR‐144 reduce hypoxia‐induced autophagy and enhance radiosensitivity of prostate cancer cells via suppressing PIM1

**DOI:** 10.1002/cam4.664

**Published:** 2016-03-14

**Authors:** Hao Gu, Mingzhu Liu, Changmao Ding, Xin Wang, Rui Wang, Xinyu Wu, Ruitai Fan

**Affiliations:** ^1^ Department of Radiation OncologyThe First Affiliated Hospital of Zhengzhou UniversityHenan450052China; ^2^ Department of Traditional Chinese MedicineThe First Affiliated Hospital of Zhengzhou UniversityHenan450052China; ^3^ Department of RadiologyThe First Affiliated Hospital of Zhengzhou UniversityHenan450052China; ^4^ Department of Nuclear MedicineHenan Provincial People's Hospital & the People's Hospital of Zhengzhou UniversityHenan450003China

**Keywords:** Autophagy, hypoxia, miR‐124, miR‐144, PIM1, radiosensitivity

## Abstract

Cancer cells in hypoxia usually make adaptive changes in cellular metabolism, such as altered autophagy. This might be a cause of enhanced radioresistance in some types of cancer. In this study, we investigated hypoxia‐responsive miRNAs in two prostate cancer cell lines (DU145 and PC3). This study firstly reported that hypoxia induces further downregulation of miR‐124 and miR‐144, which might be a result of impaired dicer expression. These two miRNAs can simultaneously target 3′UTR of PIM1. Functional study showed that miR‐124 or miR‐144 overexpression can inhibit hypoxia‐induced autophagy and enhance radiosensitivity at least via downregulating PIM1. Therefore, hypoxia induced miR‐124 and miR‐144 downregulation may contribute to a prosurvival mechanism of prostate cancer cells to hypoxia and irradiation at least through attenuated suppressing of PIM1. This finding presents a potential therapeutic target for prostate cancer.

## Introduction

Prostate cancer is one of the most common forms of male cancer and also a leading cause of malignancy‐related death in men 1. Radiotherapy is an important adjuvant treatment after prostate tumor resection and is an optional therapeutic strategy for regionally unresectable prostate tumor 2. Radioresistance is a major challenge in successful radiotherapy. In fact, prostate cancer is one of the leading radioresistant malignancies currently 3. Therefore, it is urgent to investigate the mechanisms of radioresistance and associated signaling pathways for further improvement of therapeutic strategies.

There are emerging evidence showed hypoxia is positively correlated with tumor aggressiveness and clinical stage of prostate cancer 3. In addition, hypoxia can also induce dynamic changes in prostate cancer cells, leading to increased radiation resistance 4. Some recent studies suggest that hypoxia‐responsive miRNAs might be involved in regulation of radiosensitivity of cancer cells through multiple mechanisms. For example, knockdown of miR‐210 decreases stemness and radioresistance of hypoxic glioma stem cells 5. Hypoxia induced upregulation of miR‐21 further enhances expression of HIF‐1*α* and vascular endothelial growth factor (VEGF), leading to increased tumor angiogenesis and decreased cell apoptosis after irradiation 6.

Hypoxia induced higher level of autophagy is also considered as a mechanism of radioresistance in some types of solid cancer, such as breast cancer 7 and colon cancer 8. Actually, miRNAs can also affect radiosensitivity via modulating autophagy 9, 10. For instance, miR‐200c can suppress autophagy and decrease radioresistance of breast cancer cells via targeting UBQLN1 10. The PIM kinases represent a family of serine/threonine kinases. Some recent studies suggest that the PIM kinases are involved in regulating cell cycle, apoptosis, stem cells, metabolism, and autophagy of cancer cells, thereby playing vital roles in cancer development 11, 12.

In this study, we found that miR‐124 and miR‐144 are two hypoxia‐responsive miRNAs, which can reduce hypoxia‐induced autophagy and enhance radiosensitivity of prostate cancer cells via reducing PIM1.

## Materials and Methods

### Cell culture

Prostate cancer cell lines DU145 and PC3 were obtained from American Type Culture Collection (ATCC) and were cultured in RPMI‐1640 medium supplemented with 10% fetal bovine serum, 100 U/mL penicillin, and 100 mg/mL streptomycin. The cells were maintained in a cell incubator with a humidified air containing 5% CO_2_ at 37°C. For hypoxic culture, oxygen supply was set to 2%. To quantify the change of miR‐124 and miR‐144 induced by hypoxia, DU145 and PC3 cancer cells were subjected to hypoxia up to 72 h. The expression of miR‐124 and miR‐144 at indicating time points was quantified using qRT‐PCR analysis. Variation in Dicer at indicating time points was measured using western blot analysis.

### Reagents and cell treatment

MiR‐124, miR‐144 mimics, and the scrambled negative controls were purchased from RiBoBio (Shanghai, China). Dicer and PIM1 siRNA was purchased from Santa Cruz Biotech (Santa Cruz, CA). 3‐methyladenine (3‐MA) was purchased from Sigma‐Aldrich (St Louis, MO). A pEZ‐M02‐PIM1 expression vector in which the 3′UTR of PIM1 was modified (without miR‐124 or miR‐144 specific binding sites) was synthesized by Genepharma (Shanghai, China). Transfection was performed using Lipofectamine 2000 reagent (Invitrogen) according to the manufacturer's protocol.

DU145 and PC3 cells were transfected with 100 nmol/L Dicer siRNA. MiR‐124 and miR‐144 expression was examined 48 h after transfection. To examine the influence of miR‐124 or miR‐144 on autophagy, DU145 and PC3 cells were plated in six‐well plates at 4 × 10^5^ cells/well and were transfected with 100 nmol/L miR‐124 or miR‐144 mimics. 48 h after transfection, the cells were subjected to hypoxia for 48 h or subjected to irradiation using 6 MV X‐ray generated by a linear accelerator (Varian 2300EX, Varian, Palo Alto, CA) at a dose rate of 5 Gy per min. DU145 and PC3 cells without miR‐124 or miR‐144 overexpression were treated with 3‐MA (5 mmol/L) 1 h before hypoxia or 1 h before irradiation, for a duration of 48 h. Then, the cells were subjected to western blot analysis of LC3B and p62 or subjected to clonogenic assay and flow cytometry analysis of apoptosis and western blot analysis of active caspase‐3.

To examine the functional role of PIM1, DU145 and PC3 cells were transfected with pEZ‐M02‐PIM1 expression vector alone or in combination with miR‐124 (100 nmol/L) or miR‐144 mimics (100 nmol/L). Forty‐eight hours after transfection, the cells were subjected to hypoxia for another 48 h or irradiation. Then, western blot analysis, clonogenic assay, and flow cytometry analysis were carried out.

### MiRNA microarray

Briefly, the cancer cells were subjected to normoxic or hypoxic culture for 48 h and the cells were collected. Total miRNAs in the cell samples were extracted using the miRVana miRNA Isolation Kit (Ambion, Austin, TX). Three pairs of total miRNA samples of normoxia or hypoxia cultured DU145 and PC3 cells were used for miRNA microarray analysis. Briefly, the miRNAs were labeled using the miRCURY Hy3/Hy5 Power labeling kit (Exiqon, Vedbaek, Denmark) and then hybridized on the miRCURYTM LNA microRNA Array (v.14.0) (Exiqon) according to the array manual. Then, the microarrays were scanned with the Axon GenePix 4000B microarray scanner (Axon Instruments, Foster City, CA). The scanned images were imported into GenePix Pro 6.0 software (Axon Instruments) for grid alignment and data extraction. After background subtraction and signal normalization, the miRNAs with at least fivefold difference between normoxia and hypoxia groups (*P *< 0.05) were candidates for further analysis.

### QRT‐PCR analysis

Total RNA from cells samples were extracted using TRIzol reagent (Invitrogen, Carlsbad, CA). MiRNAs corresponding cDNA was synthesized with specific stem‐loop primers and the TaqMan MicroRNA Reverse Transcription Kit (Applied Biosystems; Foster City, CA). To quantify miR‐124 and miR‐144 expression, qRT‐PCR analysis was performed using TaqMan MicroRNA Assay Kit (Applied Biosystems), with U6 snRNA used as the endogenous control.

To quantify PIM1 mRNA, pri‐miR‐124, and pri‐miR‐144 expression, the first strand cDNA was synthesized using the PrimeScript RT reagent kit (TaKaRa, Dalian, China). The PCR primers for PIM1 were as follows: forward, 5′‐TCATTAGATGGTGCTTGGCCCTGA‐3′; reverse, 5′‐TGTGGAGGTGGATCTCAGCAGTTT‐3′; for pri‐miR‐124 were: forward, 5′‐ACGGGATCCTCTTATTCCATCTTCTACCC‐3′, reverse, 5′‐CGGAATTCCTGGCTCGGTCGGTCGCTC‐3′; For pri‐miR‐144 were: forward, 5′‐GCTGGGATATCATCATATACTG‐3′, reverse: 5′‐CGGACTAGTACATCATCTATACTG‐3′. QRT‐PCR was performed using SYBR Premix Ex Taq II (TaKaRa) with an ABI 7500 Sequence Detection System (Applied Biosystems). The results were calculated using the 2^−ΔΔCT^ methods.

### Western blot analysis

Cells were lysed using a lysis buffer (Beyotime, Shanghai, China) and then protein concentration was quantified using a BCA protein assay kit (Beyotime). Then the protein samples were subjected to a conventional western blot analysis. After separation on 10% SDS PAGE gel, the proteins were subsequently transferred onto PVDF membrane and blocked with 5% nonfat milk for 1 h. Membranes were probed overnight at 4°C with primary antibodies: anti‐LC3B (1:3000, ab51520, Abcam, Cambridge, MA, USA), anti‐p62/SQSTM1 (1:1000, #8025, Cell Signaling), anti‐DICER (1:200, H‐212; Santa Cruz Biotechnology), anti‐active caspase‐3 (1:1000, ab2302, Abcam), anti‐tubulin (1:1000, ab56676, Abcam), and anti‐*β*‐actin (ab189073, 1:1000, Abcam). Band signals were visualized using HRP‐linked secondary antibodies (Abcam) and the ECL Western blotting substrate (Promega, Madison, WI, USA).

### Preparation of DU145 cells with stable GFP‐LC3 expression

Briefly, DU145 cells were transfected with pSELECT‐GFP‐LC3 (Invivogen) were screened for stable clones using 250 *μ*g/mL Zeocin (Sigma‐Aldrich) up to 3 weeks. Then, the cells with stable GFP expression were transfected with 100 nmol/L miR‐124 or miR‐144 mimics. Forty‐eight hours after transfection, the cells were subjected to hypoxia for another 48 h. Then the accumulation of GFP‐LC3 puncta was captured using Olympus IX71 inverted microscope (Olympus, Tokyo, Japan).

### Flow cytometric analysis of cell apoptosis

Cell apoptosis was detected by using Annexin V‐FITC Apoptosis Detection Kit (ab14085, Abcam) and the apoptosis rates were measured by using a flow cytometer (FACSCalibur, BD Biosciences Franklin Lakes, NJ, USA).

### Clonogenic assay

After irradiation, the plates were further incubated for 10–14 days and then the cells were fixed with 10% methanol and stained with 1% crystal violet in 70% ethanol. Colonies (>50 cells) were counted under a light microscope. The curve of survival fraction was derived from the multi‐target single‐hit model: SF = 1‐(1‐exp(‐x/D0))^N.

### Dual luciferase assay

The binding sites between miR‐124 or miR‐144 and 3′UTR of PIM1 were predicted using TargetScan 6.3. Since miR‐124 has two putative binding sites, whereasmiR‐144 has one putative binding site with 3′UTR of PIM1, three pairs of wild‐type or mutant human PIM1 3′UTR sequences with flanking *SacI* and *SalI* restriction enzyme digestion sites were chemically synthesized (Table S1). Then the sequences were interested into the sites between *SacI* and *SalI* of the pmirGLO Dual‐Luciferase miRNA Target Expression Vector (Promega), respectively. The recombinant plasmids were named as pmirGLO‐PIM1‐WT1 (with miR‐124 binding site 1), pmirGLO‐PIM1‐WT2 (with miR‐124 binding site 2), pmirGLO‐PIM1‐WT3 (with miR‐144 binding site), and pmirGLO‐PIM1‐MT1, pmirGLO‐PIM1‐MT2, and pmirGLO‐PIM1‐MT3, respectively. To assess the inhibiting effects of miR‐124 or miR‐144 on luciferase expression, DU145 cells were cotransfected with 200 ng recombinant plasmids and 100 nmol/L miR‐124 or miR‐144 mimics or the negative control using Lipofectamin 2000 (Invitrogen). Twenty‐four hours after transfection, luciferase activity was analyzed using the Dual‐Luciferase Reporter Assay System (Promega). Firefly luciferase activity was normalized to that of Renilla luciferase.

### Statistical analysis

Experimental data are presented as mean ± SD with at least three repeats, with each one performed in triplicate. One‐way ANOVA was performed to compare means of multiple group experiments. Comparison between groups was performed by using nonpaired Student's *t* test. *P* value of <0.05 was considered significant.

## Results

### Hypoxia induces significantly downregulated miR‐124 and miR‐144 in prostate cancer cells via impaired Dicer expression

**Figure 1:**
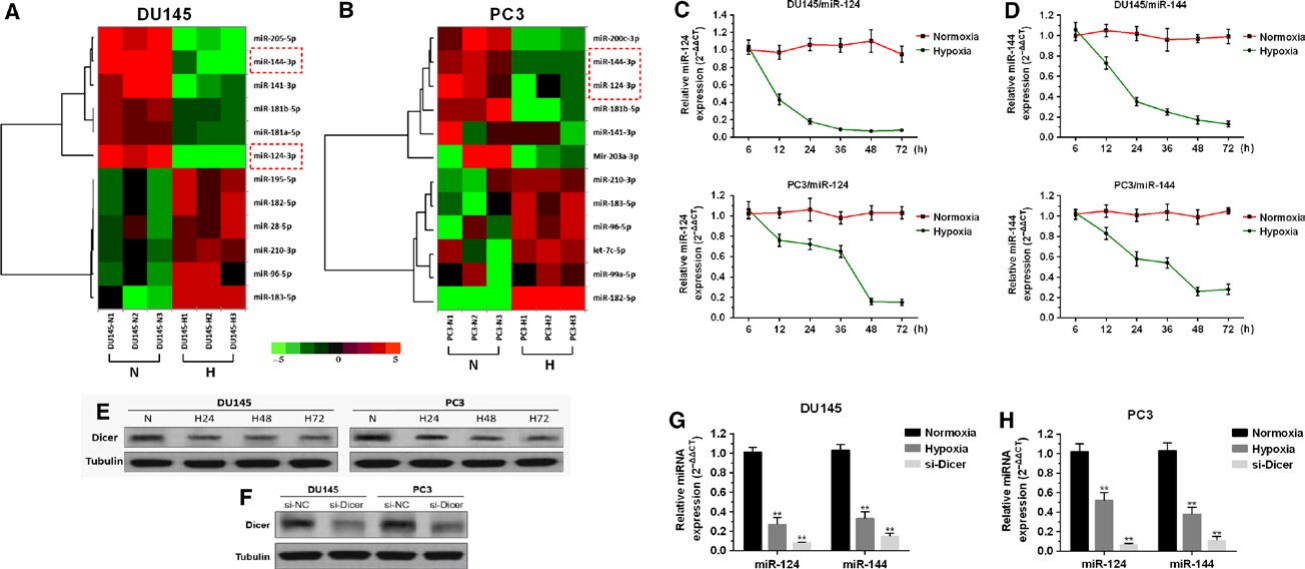
Hypoxia induces significant downregulation of miR‐124 and miR‐144 in prostate cancer cells via impaired Dicer expression (A and B) 12 mostly changed miRNAs (6 upregulated and 6 downregulated) between normoxia and hypoxia cultured DU145 (A) and PC3 (B) cells were identified according to the criteria of fold change≥5, *P *< 0.05. Columns represent samples and rows represent miRNAs (black, green, and red correspond to unchanged, downregulated and upregulated, respectively; H: hypoxia, N: normoxia). (C and D) QRT‐PCR analysis of miR‐124 (C) and miR‐144 (D) expression in normoxic and hypoxic cultured DU145 and PC3 cells at indicating time points up to 72 h. (E and F) Western blot analysis of Dicer in normoxic and hypoxic cultured DU145 and PC3 cells at indicating time points up to 72 h (E) and in the cells 48 h after si‐Dicer transfection (F). H24/H48/H72 = 24/48/72 h post hypoxia. (G and H) The ratio of mature miRNA to pri‐miRNA for miR‐124 and miR‐144 after hypoxia or Dicer knockdown in DU145 (G) and PC3 (H) cells. Mature and pri‐miRNA levels were determined by qRT‐PCR. * *P < *0.05, ** *P < *0.01.

Hypoxia can induce dysregulated expression of miRNAs in prostate cancer cells 13. By comparison of miRNA expression in DU145 and PC3 cells after hypoxic or normoxic culture, we observed that multiple miRNAs were significantly downregulated in hypoxia (Fig. 1A and B). MiR‐124 and miR‐144 are two tumor suppressors of prostate cancer 14, 15, 16, 17. The microarray analysis showed these two miRNAs were decreased dramatically in hypoxia in both DU145 and PC3 cells (Fig. 1A and B). Following qRT‐PCR analysis confirmed the expression of miR‐124 (Fig. 1C) and miR‐144 (Fig. 1D) in DU145 and PC3 cells gradually declined under hypoxic culture. One recent study showed that hypoxia can induce epigenetic regulation of Dicer, leading to silencing of the Dicer promoter and subsequently decrease in Dicer expression 18. Thus, we hypothesized that hypoxia induced miR‐124 and miR‐144 decrease might be related to dysregulated Dicer. By performing western blot analysis, we found that Dicer expression at protein level substantially decreased in hypoxia (Fig. 1E). Then, we knocked down endogenous Dicer in both DU145 and PC3 cells (Fig. 1F). Both DU145 and PC3 cells with Dicer knockdown had significantly lower ratio of mature miRNA to pri‐miRNA for both miR‐124 and miR‐144 (Fig. 1G and H), suggesting a defect in miRNA processing caused by Dicer repression. These results suggested that hypoxia induces significant downregulation of miR‐124 and miR‐144 in prostate cancer cells, which might be related to decreased Dicer expression.

### MiR‐124 and miR‐144 reduce hypoxia‐induced autophagy and enhance radiosensitivity of prostate cancer cells

**Figure 2:**
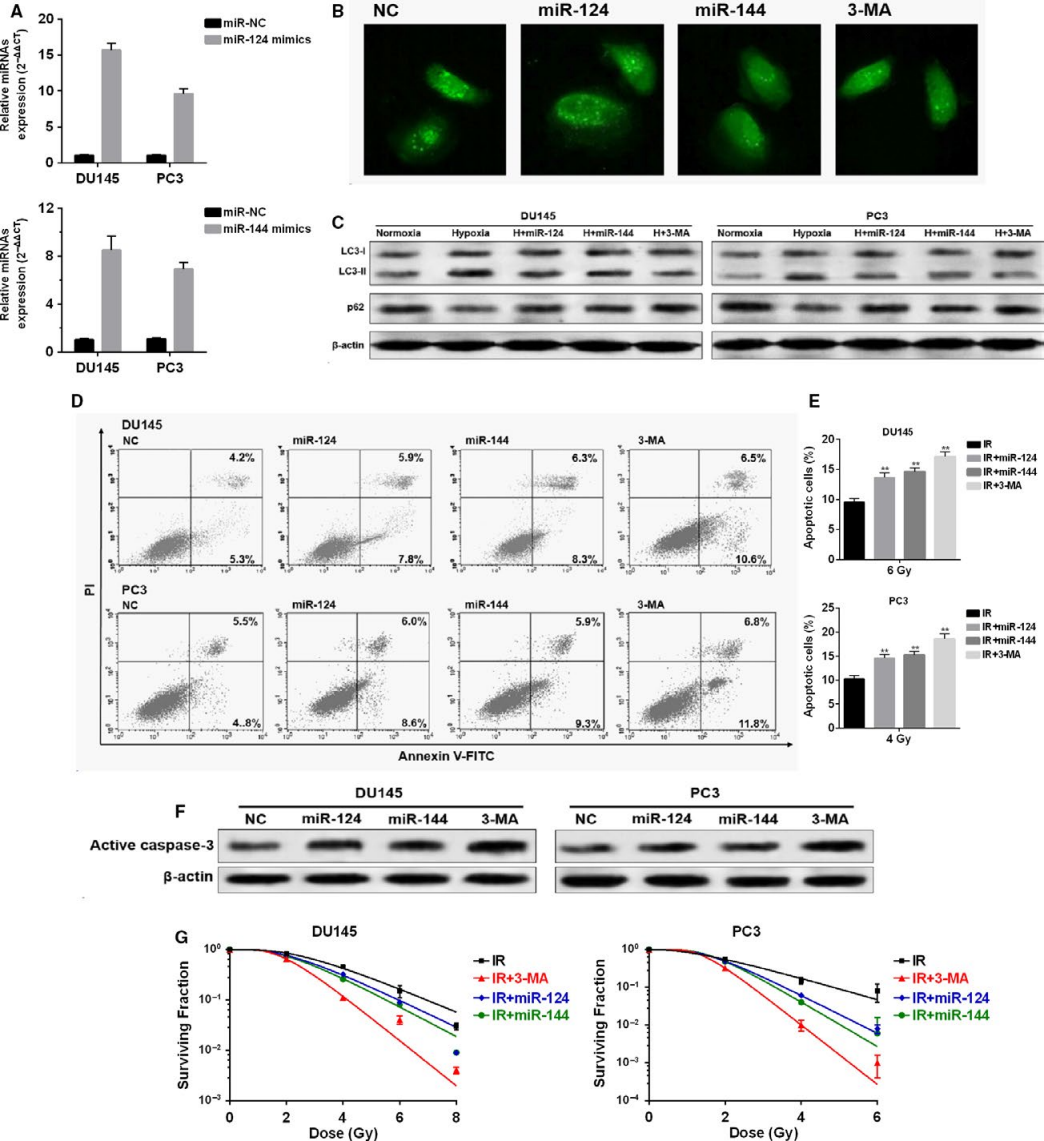
MiR‐124 and miR‐144 reduce hypoxia‐induced autophagy and enhance radiosensitivity of prostate cancer cells (A) QRT‐PCR analysis of miR‐124 and miR‐144 expression in DU145 and PC3 cells transfected with miR‐124 or miR‐144 mimics. (B) DU145 cells with stable GFP‐LC3 expression were then transfected with a miR‐124 or miR‐144 mimics. Forty‐eight hours after transfection, the transfected cells and the cells treated with 3‐MA were subjected to hypoxia for another 48 h. Then the accumulation of GFP‐LC3 puncta was captured using a fluorescence microscope. (C) Western blot analysis of LC3 and p62 in DU145 and PC3 cells in normoxia or with the same treatment as in figure B. (D) Flow cytometry analysis of apoptotic DU145 or PC3 cells with indicating treatments after irradiation. (E) Quantification of the ratio of apoptotic cells. (F) Western blot analysis of active caspase‐3 in DU145 and PC3 cells with indicating treatments after irradiation. (G) Survival fraction of DU145 and PC3 cells with indicating treatments after irradiation. * *P < *0.05, ** *P < *0.01.

Increased autophagy usually acts a prosurvival mechanism of cancer cells under hypoxia and also might be an important mechanism of enhanced survival after irradiation 10. To investigate the effect of miR‐124 and miR‐144 on autophagy and radiosensitivity, DU145 and PC3 cells were firstly transfected with miR‐124 or miR‐144 mimics for overexpression (Fig. 2A). In DU145 cells, we observed that miR‐124 or miR‐144 overexpression significantly alleviated accumulation of GFP‐LC3 puncta in DU145 cells in hypoxia, the effect of which was quite similar to 3‐MA, an autophagy inhibitor (Fig. 2B). Following western blot analysis confirmed that miR‐124 or miR‐144 overexpression attenuated hypoxia induced higher expression of LC3‐II and p62 degradation (Fig. 2C). These results suggested that both miR‐124 and miR‐144 can reduce hypoxia‐induced autophagy. Then, we explored the involvement of these two miRNAs in radioresistance of prostate cancer cells. In both DU145 and PC3 cells, miR‐124 or miR‐144 overexpression increased the rate of apoptotic cells after irradiation (Fig. 2D and E). Also miR‐124 or miR‐144 overexpression enhanced irradiation‐induced activation of caspase‐3 (Fig. 2F) and reduced colonies formed after irradiation (Fig. 2G).

### MiR‐124 and miR‐144 simultaneously target 3′UTR of PIM1 and decrease its expression

**Figure 3:**
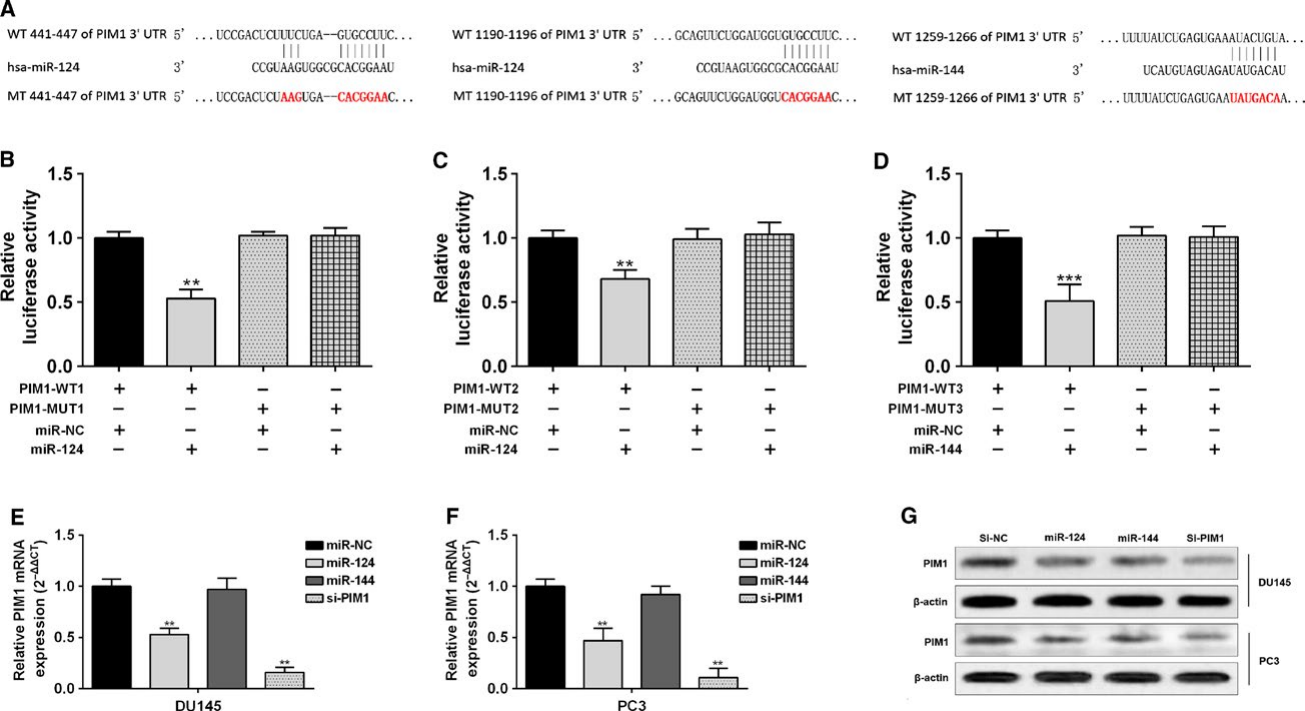
MiR‐124 and miR‐144 simultaneously target 3′UTR of PIM1 and decrease its expression (A) Predicted binding sites between miR‐124 or miR‐144 and 3′UTR of PIM1. The designed mutant sequence was also given. (B–D) Dual luciferase assay of the inhibitive effect of miR‐124 or miR‐144 on relative luciferase activity in DU145 cells transfected miR‐124 or miR‐144 mimics and the reporters carrying either wild‐type or mutant sequences. (E–G) QRT‐PCR (E, F) and western blot (G) analysis of PIM1 mRNA (E, F) and protein (G) expression in DU145 or PC3 cells after transfection with miR‐124 or miR‐144 mimics or PIM1 siRNA. * *P < *0.05, ** *P < *0.01, *** *P < *0.001.

By performing prediction using TargetScan 6.3, we observed that miR‐124 and miR‐144 are both potential regulators of PIM1, a well‐recognized oncogene of prostate cancer 19. Online prediction showed that miR‐124 has two putative binding sites, whereas miR‐144 had one putative binding site with 3′UTR of PIM1 (Fig. 3A). By performing dual luciferase assay, we found both miR‐124 and miR‐144 could effectively bind to the predicted binding sites, which was reflected by the decreased luciferase activity of reporters with wild‐type PIM1 3′UTR sequence (Fig. 3B–D). In both DU145 and PC3 cells, enforced miR‐124 expression significantly decreased PIM1 expression at both mRNA and protein level (Fig. 3E–G). Although miR‐144 could not promote degradation of PIM1 mRNA(Fig. 3E and F), it substantially suppressed PIM1 protein expression (Fig. 3G). These results suggest that miR‐124 and miR‐144 can simultaneously target 3′UTR of PIM1 and reduce its expression in prostate cancer cells.

### MiR‐124 and miR‐144 reduce hypoxia‐induced autophagy and enhance radiosensitivity of prostate cancer cells via PIM1

**Figure 4:**
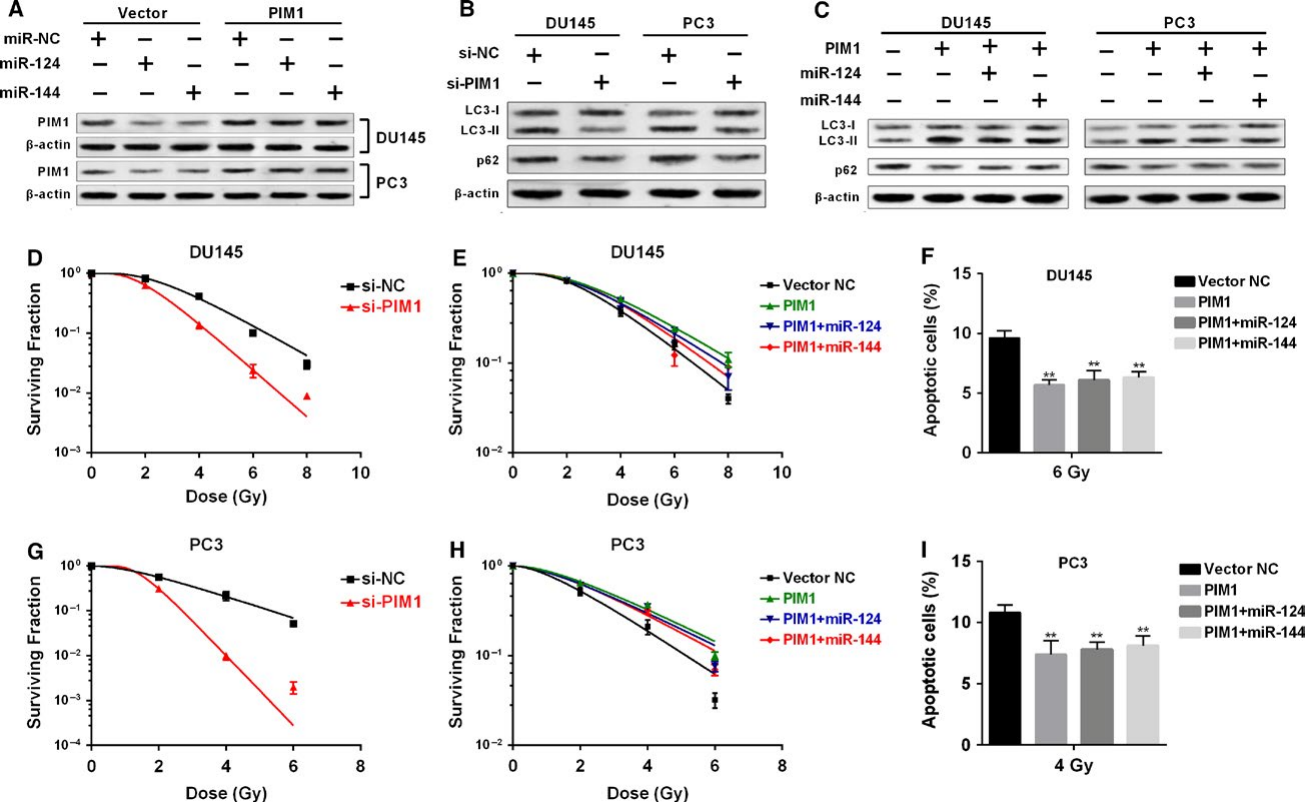
MiR‐124 and miR‐144 reduce hypoxia‐induced autophagy and enhance radiosensitivity of prostate cancer cells via PIM1 (A) Western blot analysis of PIM1 expression in DU145 and PC3 cells transfected with PIM1 expression vector alone or in combination with miR‐124 or miR‐144 mimics. (B, C) Western blot analysis of LC3 and p62 expression in DU145 and PC3 cells with or without PIM1 knockdown (B) or with PIM1 overexpression alone or in combination with miR‐124 or miR‐144 mimics (C) 48 h after hypoxia. (D, E, G, and H) Survival fraction of DU145 and PC3 cells with transfection described in figure B (D, E) or figure C (G, H). (F and I) Quantification of flow cytometry analysis of irradiation‐induced apoptotic DU145 (F) and PC3 (I) cells with transfection described in figure (C) * *P < *0.05, ** *P < *0.01.

Since we confirmed the regulative effect of miR‐124 and miR‐144 on PIM1, we then investigated their functional role in hypoxia‐induced autophagy and radiosensitivity of prostate cancer cells. We firstly overexpressed PIM1 in DU145 and PC3 cells using a recombined vector with mutant miR‐124 and miR‐144 binding sites (Fig. 4A). MiR‐124 or miR‐144 overexpression had no effect on PIM1 expression of this vector in both cell lines (Fig. 4A). Knockdown of PIM1 significantly attenuated hypoxia‐induced autophagy (Fig. 4B), whereas PIM1 overexpression substantially enhanced hypoxia‐induced autophagy (Fig. 4C). Knockdown of PIM1 significantly decreased cell survival after irradiation (Fig. 4D and G). But enforced PIM1 expression remarkably promoted cell survival and reduced cell apoptosis after irradiation in both DU145 (Fig. 4E and F) and PC3 cells (Fig. 4H and I). However, neither miR‐124 nor miR‐144 could suppress the effects of this expression vector on autophagy, cell survival, and apoptosis.

## Discussion

Hypoxia is an important tumor microenvironment stressor forcing cancer cells make adaptive responses. Hypoxia‐responsive miRNAs may participate in multiple pathological processes of the cancer. For instance, hypoxia induced miR‐96 expression can stimulate autophagy via inhibiting MTOR in prostate cancer cells 20. HIF1*α* induced upregulation of miR‐182 can enhance HIF1 signaling via suppressing the expression of PHD and FIH1, two negative regulators of HIF1 13. In addition, hypoxia can also induce upregulation of miR‐21 in prostate cancer, which has wide influences on cell cycle, DNA damage repair, apoptosis, and autophagy of cancer by targeting different genes 6.

Although the tumor suppressive effects of miR‐124 and miR‐144 were reported in previous studies 14, 15, 16, 17, whether their expression was altered in hypoxia has not been reported yet. In this study, we observed that miR‐124 and miR‐144 were significantly downregulated in hypoxia, which might be a result of impaired Dicer expression. In addition, by overexpressing miR‐124 or miR‐144 in DU145 and PC3 cells, we found these two miRNAs suppressed autophagy. Autophagy is an important prosurvival mechanism of prostate cancer cells in hypoxia and androgen deprivation 21, 22. Inhibition of autophagy directly resulted in increased prostate cancer apoptosis in hypoxia 23. In this study, we found that enforced miR‐124 and miR‐144 expression enhanced radiosensitivity and apoptosis in both DU145 and PC3 cells, the effects of which were quite similar to 3‐MA, an autophagy inhibitor. Therefore, inhibiting autophagy might be a potential strategy to enhance radiosensitivity when giving radiotherapy to prostate cancer.

Then, we decided to further investigate the underlying mechanisms. The PIM kinases have a wide range of regulation on proliferation, apoptosis, cell cycle, and angiogenesis of cancer 19. PIM1 expression is usually upregulated in prostate cancer, which could be a result of activation of the JAK/STAT pathway 19. PIM1 mRNA and protein production are further induced under hypoxic conditions 24. But the mechanism has not been fully revealed. One previous study found that in hypoxia, heat shock protein 90 (HSP90) can stabilize PIM1, thus preventing PIM1 degradation 25. Through online prediction, we observed that miR‐124 and miR‐144 might simultaneously target PIM1. By performing dual luciferase assay, we confirmed the binding between miR‐124 or miR‐144 and PIM1 3′UTR. Through qRT‐PCR and western blot analysis, we confirmed the regulative effect of miR‐124 and miR‐144 on PIM1 expression. Therefore, the hypoxia induced lower miR‐124 and miR‐144 expression might also be a contributor of increased PIM1 expression in hypoxia. Functionally, PIM1 can cooperate with MYC and promote prostate tumorigenesis 19. Besides, it can phosphorylate of androgen receptor and facilitate its degradation 19. In addition, PIM1 may also exert protective effect on prostate cancer cells to chemotherapeutic drugs. One recent study found that enforced expression of PIM1 protected RWPE‐2 and DU145 cells from docetaxel‐induced cell death. However, down‐regulation of endogenous PIM1 reduced cell viability and enhanced apoptosis of the cells after docetaxel treatment 12. However, few studies investigated the impact of PIM1 on autophagy and radioresistance of prostate cancer. In this study, we found PIM1 overexpression enhanced hypoxia‐induced autophagy and reduced apoptosis after irradiation. In contrast, knockdown of endogenous PIM1 reduced autophagy induced by hypoxia and sensitized prostate cancer cells to irradiation. These results suggest that the PIM1 is an important gene modulating adaptive responses of prostate cancer cells to environmental stressors, such as hypoxia and radiation.

## Conclusion

In conclusion, this study firstly reported that hypoxia can induce further downregulation of miR‐124 and miR‐144, which simultaneously target 3′UTR of PIM1. MiR‐124 or miR‐144 overexpression can inhibit hypoxia‐induced autophagy and enhance radiosensitivity at least via downregulating PIM1 in prostate cancer cells. Therefore, hypoxia‐induced miR‐124 and miR‐144 downregulation may contribute to a prosurvival mechanism of prostate cancer cells to hypoxia and irradiation at least through attenuated suppressing of PIM1. This presents a potential therapeutic target for prostate cancer.

## Conflict of Interest

None Declared.

## Supporting information


**Table S1.** The wild‐type and mutant binding sites between miR‐124 or miR‐144 and 3′UTR of PIM1
