# Supplementary material for: Hypoxia‐responsive miR‐124 and miR‐144 reduce hypoxia‐induced autophagy and enhance radiosensitivity of prostate cancer cells via suppressing PIM1
Source: Cancer Med. 2016 Mar 14;5(6):1174–82. doi: 10.1002/cam4.664 (PMC4924376; doi:10.1002/cam4.664)
Supplement: Supplementary file 1 — Table S1. The wild‐type and mutant binding sites between miR‐124 or miR‐144 and 3′UTR of PIM1 [file CAM4-5-1174-s001.docx]

**Supplementary table 1 The wild-type and mutant binding sites between miR-124 or miR-144 and 3’UTR of PIM1**

**MiR-124 binding site 1**

WT1, F, 5’-cCTCTTCCGACTCTTTCTGAGTGCCTTCTGTGGGGACTCCGGCTGTGCTGGGAGAg-3’,

R,5’-tcgacTCTCCCAGCACAGCCGGAGTCCCCACAGAAGGCACTCAGAAAGAGTCGGAAGAGgagct-3’

MT1, F, 5’-cCTCTTCCGACTCTAAGTGACACGGAACTGTGGGGACTCCGGCTGTGCTGGGAGAg-3’,

R,5’-tcgacTCTCCCAGCACAGCCGGAGTCCCCACAGTTCCGTGTCACTTAGAGTCGGAAGAGgagct-3’

**MiR-124 binding site 2**

WT2, F, 5’-cATGAAAAGCAGTTCTGGATGGTGTGCCTTCCAGATCCTCTCTGGGGCg-3’

R,5’-tcgacGCCCCAGAGAGGATCTGGAAGGCACACCATCCAGAACTGCTTTTCATgagct-3’

MT2, F, 5’-cATGAAAAGCAGTTCTGGATGGTCACGGAACCAGATCCTCTCTGGGGCg-3’

R, 5’-tcgacGCCCCAGAGAGGATCTGGTTCCGTGACCATCCAGAACTGCTTTTCATgagct-3’

**MiR-144 binding site**

WT3,F, 5’-cGGTAGCCTGCTGGTTTTATCTGAGTGAAATACTGTACAGGGGAATAAAAGAGATCTTAg-3’

R, 5’-tcgacTAAGATCTCTTTTATTCCCCTGTACAGTATTTCACTCAGATAAAACCAGCAGGCTACCgagct-3’

MT3,F, 5’-cGGTAGCCTGCTGGTTTTATCTGAGTGAATATGACAACAGGGGAATAAAAGAGATCTTAg-3’

R, 5’-tcgacTAAGATCTCTTTTATTCCCCTGTTGTCATATTCACTCAGATAAAACCAGCAGGCTACCgagct-3’
